# Editorial: Integrative Approaches to the Molecular Physiology of Inflammation, Volume II

**DOI:** 10.3389/fphys.2022.873295

**Published:** 2022-03-18

**Authors:** Enrique Hernández-Lemus, Carlos Rosales, María Elena Soto

**Affiliations:** ^1^Computational Genomics Division, National Institute of Genomic Medicine (INMEGEN), Mexico City, Mexico; ^2^Centro de Ciencias de la Complejidad, Universidad Nacional Autónoma de México, Mexico City, Mexico; ^3^Instituto de Investigaciones Biomédicas, Universidad Nacional Autónoma de México, Mexico City, Mexico; ^4^Department of Immunology, National Institute of Cardiology Ignacio Chávez, Mexico City, Mexico

**Keywords:** inflammation, molecular physiology, integrative biology, immune responses, systemic inflammation

In the second volume of the Integrative Approaches to the Molecular Physiology of Inflammation, issues covered converge to themes such as the role of low-grade inflammation and long-term sustained inflammatory processes in the development of systemic pathologies, how do bimolecular regulation processes may shape signaling and metabolic pathways leading to inflammatory responses and even how these same phenomena may be behind COVID-19 pathophysiology. The volume also presents an interesting balance between original research results and comprehensive review articles. We believe that such balance is relevant in shaping a representative overview of the issues covered.

It has been recently debated that low-grade inflammation (LGI), originally considered to be a source of somehow mild pathological consequences, may have indeed rather severe consequences. However, LGI is quite difficult to diagnose and often this condition is not discovered until discernible symptoms are present, commonly appearing once disease processes are on advanced stages, bringing about health-damaging consequences. Developing LGI biomarkers (particularly those serving as ‘early-warnings') for several conditions and tissues is a desirable but challenging endeavor. The original research article by van Bilsen et al. is a relevant contribution along these lines. It presents a mechanism-based method to predict LGI in the liver and adipose tissue. The authors used a computational (*in silico*) approach to select for candidate biomarkers based on previously known mechanistic knowledge contained in large public databases such as the Gene Ontology and Human Protein Atlas. Those candidate biomarkers were further validated a murine model of fat-diet induced LGI with posterior transcriptome analysis of the liver and adipose tissue and their results were later assessed in a customized PubMed database search.

The organismal level effects of LGI and other systemic conditions on human health are thoroughly discussed in the review article by Martínez-García and Hernández-Lemus in the context of the plethora of diseases associated with periodontal inflammation. The authors present a panoramic view comprising the biological aspects of periodontal inflammation staring from its infectious pathogenesis, the associated cellular and tissular processes, immune and signaling pathways involved, as well as the genetic and epigenetic phenomena contributing to the susceptibility, onset and systemic dissemination of periodontal-related inflammatory processes. The medical and clinical aspects related to the comorbidities of periodontal disease, being those chronic ailments such as cancer, metabolic conditions, cardiovascular, neurologic and respiratory diseases; autoimmune and systemic inflammation conditions and even COVID-19 complications. Those aspects are discussed in the context of the immunological and signaling pathways that have been reported in the recent literature on these subjects.

The review by Shi et al., discusses the epigenetic regulation of stellate cells and macrophages and the role this regulation has in liver inflammation. It is well known that moderate liver inflammation can protect the liver from damage and facilitate the recovery of liver injury. However, when inflammation is too intense and becomes chronic, it can lead to massive death of hepatocytes and to irreversible damage to the liver parenchyma. Under different stress conditions, stellate cells and macrophages actively participate in promoting inflammation. This review explores the mechanisms of epigenetics that regulate liver inflammation, and proposes new ideas for therapy of liver diseases.

Molecular processes related to the immune-mediated inflammatory phenotypes can be unveiled by high throughput omic studies. This is the case shown in the contribution by Pinto, et al. They used a comparative proteomic analysis to evaluate the varying impact on immune responses presented in a drug-mediated (Phorbol 12-Myristate-13-Acetate, PMA) THP-1 monocyte-to-macrophage differentiation model. By resorting to dimethyl labeling-based quantitative proteomics they were able to determine how the protein repertoire of macrophage-like cells differentiated from THP-1 monocytes changes. Furthermore, the authors developed an integrated network analysis to evidence variations in PMA concentration as well as evaluate the duration of rest post-stimulation leading to downstream differences in protein expression and, consequently, also on cellular signaling processes. Such variations were consistent with measured altered inflammatory responses, such as variation in cytokine expression upon stimulation with TLR agonists.

In relation to the role that inflammation and immune responses can play on systemic alterations related to SARS-CoV-2 infection, this volume has a couple of contributions. The way that lipid profile alterations and abnormal desaturases activity affect patients with severe pneumonia induced by SARS-CoV-2 infection is described by Pérez-Torres et al. in their original research article. On their hybrid clinic and basic study, they analyzed patients, 18 years and older admitted to a COVID-19 ward ICU who have or have not developed septic shock secondary to pneumonia, and studied their lipid profiles founding altered metabolism. Metabolic changes included a decrease in palmitic and stearic acids, increase in oleic and arachidonic acids, among others. These alterations may play a dual role: on the one hand these may favor viral replication, while on the other hand contribute to anti-viral defense by providing enhanced repair of damaged cell membranes.

Also, in connection with the metabolic and systemic effects of COVID-19 is the discussion raised by the mini review by Di Maro et al. that presents a brief by panoramic view of the way the renin angiotensin system and the cholinergic anti-inflammatory pathway modulate lung inflammation in patients with COVID-19, as well as the crosstalk and downstream interactions among these pathways that enhance a series of processes triggering anti-inflammation in the context of SARS-CoV-2 infection.

In conclusion, the studies published in Volume II of this Research Topic continue providing relevant clues helping us to understand better the molecular physiology of inflammation.

## Author Contributions

All authors listed have made a substantial, direct, and intellectual contribution to the work and approved it for publication.

## Conflict of Interest

The authors declare that the research was conducted in the absence of any commercial or financial relationships that could be construed as a potential conflict of interest.

## Publisher's Note

All claims expressed in this article are solely those of the authors and do not necessarily represent those of their affiliated organizations, or those of the publisher, the editors and the reviewers. Any product that may be evaluated in this article, or claim that may be made by its manufacturer, is not guaranteed or endorsed by the publisher.

